# Combined association of lipoprotein(a) and European Society of Cardiology Systematic COronary Risk Evaluation 2 (SCORE2) with 10-year major adverse cardiovascular events: evidence from a single tertiary hospital including 9979 patients

**DOI:** 10.1093/ehjopen/oeaf048

**Published:** 2025-04-30

**Authors:** Kyuwoong Kim, Minkyoung Kim, Jiye Han, Tae Joon Jun, Young-Hak Kim

**Affiliations:** National Cancer Control Institute, National Cancer Center, 323 Ilsan-ro, Ilsandong-gu, Goyang 10408, Republic of Korea; Graduate School of Cancer Science and Policy, National Cancer Center, 323 Ilsan-ro, Ilsandong-gu, Goyang 10408, Republic of Korea; Department of Information Medicine, Asan Medical Center, 88, Olympic-ro 43-gil, Songpa-Gu, Seoul 05505, Republic of Korea; Department of Information Medicine, Asan Medical Center, 88, Olympic-ro 43-gil, Songpa-Gu, Seoul 05505, Republic of Korea; Department of Medical Informatics and Statistics, Asan Medical Center, University of Ulsan College of Medicine, 88, Olympic-ro 43-gil, Seoul 05505, Republic of Korea; Divison of Cardiology, Department of Internal Medicine, Asan Medical Center, 88, Olympic-ro 43-gil, Seoul 05505, Republic of Korea

## Introduction

In a recent update, the European Society of Cardiology (ESC) introduced Systematic COronary Risk Evaluation (SCORE)2, a new risk prediction algorithm for fatal and non-fatal cardiovascular disease (CVD) for the European population aged between 40 and 69 without prior CVD history or diabetes.^1^ Notably, the new ESC SCORE2 chart utilizes non-HDL instead of total cholesterol compared with the previous model to address simplification, presentation, and regional variations.^1,2^ Meanwhile, lipoprotein(a) [Lp(a)], a lipoprotein particle carrying a cholesterol molecule, has been widely recognized an emerging and independent risk factor for CVD beyond the established markers considered in SCORE2.^3–7^ Therefore, we investigated the combined association of Lp(a) and ESC SCORE2 with 10-year major adverse cardiovascular events (MACE) using population-based data.

## Methods

We identified patients who were enrolled at the Asan Medical Center (Seoul, Republic of Korea) due to confirmed or suspected symptoms of unstable angina, stable angina, asymptomatic coronary artery disease (CAD), transient ischaemic attack (TIA), and peripheral artery disease (PAD) and were aged 40–69 years old without diabetes. Among these patients, we included those who were screened for Lp(a) between 1 January 2000 and 31 December 2020 with verified records on age, sex, cigarette smoking status, blood pressure, and total and HDL cholesterol. Plasma Lp(a) levels were measured using an immunonephelometric assay (BN II, Behring, Germany) calibrated against internal reference materials traceable to Siemens Health Diagnostics. Lipoprotein(a) concentrations were expressed in milligrams per decilitre (mg/dL) and subsequently categorized into quintiles (Q), with Q1–4 grouped together and compared against Q5. Additionally, Lp(a) levels were analysed based on values above and below the median.

Considering the CVD mortality rate in the Republic of Korea, which falls within the ESC SCORE2-moderate-risk region (100 to <150 CVD deaths per 100 000 population), we adopted the SCORE2 risk assessment tool. To assess the combined association of Lp(a) with ESC SCORE2, we created the following categories: Lp(a) Q1–4 and SCORE2 low or moderate risk, Lp(a) Q5 and SCORE2 low or moderate risk, Lp(a) Q1–4 and SCORE2 high risk, and Lp(a) Q5 and SCORE2 high. Additionally, analyses were conducted using Lp(a) values below the median in place of Q1–4 and values above the median in place of Q5. In this study, the patients were followed up to 10 years from the index date (i.e. first date on Lp(a) screening and assessment of SCORE2) to the first date of MACE [i.e. myocardial infarction (ICD-10: I21–I23) with history of coronary angiography or percutaneous coronary intervention, ischaemic stroke (ICD-10: I63) confirmed by brain imaging, or death from CVD or other causes identified from electronic medical records with validation from at least two specialists) or the end of the follow-up period (31 December 2020).

We used cumulative incidence function curve to visually compare the incidence of MACE among the four Lp(a) and SCORE2 groups. We also used Cox proportional hazards model adjusted for body mass index, atrial fibrillation, chronic kidney disease, subtypes of atherosclerotic CVD, screening year for Lp(a), statins, and other lipid-lowering drugs to estimate hazard ratios (HRs) and 95% confidence intervals (CIs) for MACE according to Lp(a) and SCORE2 groups.

All data collection, data visualization, and statistical analyses were conducted with R version 4.3.3 (Angel Food Cake) and SAS 9.4 (SAS Institute, Cary, NC, USA). Statistical significance was assessed using two-sided tests with a threshold of *P* < 0.05. This study was approved by the Institutional Review Board (IRB) at Asan Medical Center, Seoul, Republic of Korea (IRB no.: 202310010231001).

## Results

The study population comprised 9979 patients (mean age 58.2 years, 26.3% women, 24.7% smokers, mean systolic blood pressure 124.8 mmHg, mean non-HDL cholesterol 120.5 mg/dL). Median and interquartile range (IQR) across Q1, Q2, Q3, Q4, and Q5 of Lp(a) were 5.8 mg/dL (IQR: 4.6–6.9), 11.3 mg/dL (IQR: 9.7–13.0), 19.3 mg/dL (IQR: 17.0–21.9), 32.2 mg/dL (IQR: 28.2–37.2), and 65.8 mg/dL (IQR: 52.7–85.5). The mean values of SCORE2 across Lp(a) quintiles were as follows: 6.46 for Q1, 6.47 for Q2, 6.53 for Q3, 6.58 for Q4, and 6.64 for Q5, showing a slightly increasing trend across quintiles. The most common confirmed or suspected cardiovascular conditions were stable angina (45.8%) and unstable angina (29.9%), followed by asymptomatic CAD (17.6%), TIA (5.8%), and PAD (1.9%). Among these participants, 98.9% had a single condition, 1.0% had two, and 0.01% had three. The distribution of confirmed or suspected cardiovascular conditions of the patients across quintiles of Lp(a) levels (Q1–5) demonstrated relatively consistent percentages for unstable angina (5.41–6.64%), stable angina (8.28–10.1%), asymptomatic CAD (3.34–3.76%), TIA (1.01–1.45%), and PAD (0.36–0.41%) across the quintiles, suggesting a stable pattern regardless of Lp(a) levels. Major adverse cardiovascular events were more frequently observed in individuals with higher ESC SCORE2 categories and mid-to-high Lp(a) quintiles, with the highest frequency around Quintile 3.

**Figure 1:**
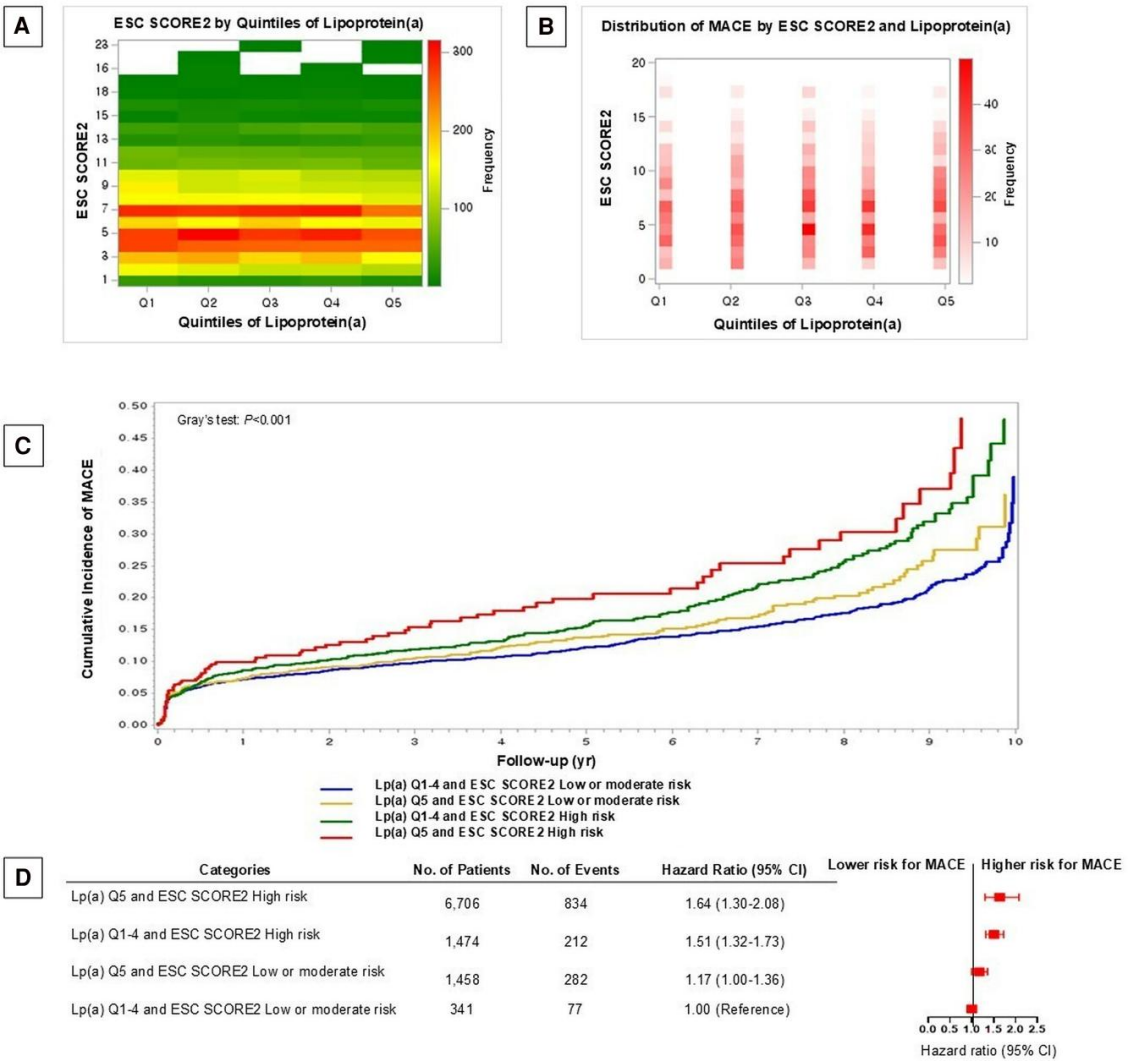
(*A*) Distribution of European Society of Cardiology Systematic COronary Risk Evaluation 2 by quintiles of lipoprotein(a) level in atherosclerotic patients. Note: Median and interquartile range for each lipoprotein(a) quintile are as follows (unit: mg/dL): Q1: 5.8 (4.6–6.9), Q2: 11.3 (9.7–13.0), Q3: 19.3 (17.0–21.9), Q4: 32.2 (28.2–37.2), and Q5: 65.8 (52.7–85.5). To convert the values for lipoprotein(a) to millimoles per litre, multiply by 2.5. (*B*) Distribution of major adverse cardiovascular events according to European Society of Cardiology Systematic COronary Risk Evaluation 2 and quintiles of liporpotein(a) level in atherosclerotic patients. (*C*) Cumulative incidence of major adverse cardiovascular events among atherosclerotic patients categorized by lipoprotein(a) level (Q1–4 and Q5) and European Society of Cardiology Systematic COronary Risk Evaluation 2 during 10 years of follow-up. (*D*) Combined association of lipoprotein(a) level and European Society of Cardiology Systematic COronary Risk Evaluation 2 with major adverse cardiovascular in atherosclerotic patients. Note: Hazard ratio and 95% confidence intervals presented above were calculated from Cox proportional hazards model adjusted for body mass index, atrial fibrillation, chronic kidney disease, subtypes of atherosclerotic cardiovascular disease (myocardial infarction, angina, asymptomatic coronary artery disease, ischaemic stroke/transient ischaemic attack, and peripheral artery disease), screening year for Lp(a), statin, and other lipid-lowering drugs. To convert the values for lipoprotein(a) to millimoles per litre, multiply by 2.5. European Society of Cardiology Systematic COronary Risk Evaluation 2 was calculated from the European Society of Cardiology Systematic COronary Risk Evaluation 2 chart for moderate region based on cardiovascular disease mortality (100 to <150 deaths from cardiovascular disease per 100 000 population). CI, confidence interval; ESC, European Society of Cardiology, Lp(a), lipoprotein(a); MACE, major adverse cardiovascular events; SCORE, Systematic COronary Risk Evaluation; No, number.

During 10 years of follow-up, a total of 1405 MACE were observed and MACE incidence was the highest in Lp(a) Q5 and SCORE2 high-risk group, followed by Lp(a) Q1–4 and SCORE2 high-risk group, Lp(a) Q5 and SCORE2 low- or moderate-risk group, and Lp(a) Q1–4 and SCORE2 low- or moderate-risk group (Gray’s test: *P* < 0.001). Compared with Lp(a) Q1–4 and SCORE2 low- or moderate-risk group, the multivariable-adjusted HRs and 95% CIs for MACE in Lp(a) Q5 and SCORE2 low- or moderate-risk group, Lp(a) Q1–4 and SCORE2 high-risk group, and Lp(a) Q5 and SCORE2 high-risk group were 1.17 (1.00–1.36), 1.51 (95% CI: 1.32–1.73), and 1.64 (95% CI: 1.30–2.08), respectively (*Figure 1*). Similar associations were found when analyses were conducted using Lp(a) values below the median in place of Q1–4 and values above the median in place of Q5, with corresponding HRs of 1.11 (95% CI: 0.98–1.26), 1.51 (95% CI: 1.27–1.79), and 1.65 (95% CI: 1.39–1.96), respectively.

## Discussion

Patients with elevated Lp(a) levels who were classified as low or moderate SCORE2 risk scores had a significantly higher risk of MACE compared with their counterparts with the same classification of SCORE2 risk scores without elevated Lp(a) levels. Those with elevated Lp(a) levels and high SCORE2 risk scores showed the highest excess risk of MACE. Our findings suggest an independent role of Lp(a) in contributing to excess risk of MACE in patients with low or moderate SCORE2 risk scores possibly owing to its proatherogenic, prothrombotic, and oxidative stress-promoting effects. Recent evidence from observational studies supports the clinical importance of screening for Lp(a) in addition to other traditional CVD risk assessments. Notably, a pooled analysis of two multiethnic cohorts in the USA showed both independent and joint associations of Lp(a) and coronary artery calcium (CAC) score with CVD risk.^8^ However, it is noteworthy that elevated Lp(a) level did not effectively stratify CVD risk in participants with no detectable calcium buildup (i.e. CAC score = 0) or low calcium buildup (CAC < 100) in the US cohorts. Taken together, whether incorporating Lp(a) measurement into well-established CVD risk assessment tools could refine risk stratification for individuals categorized as low-risk warrants further investigation.

### Limitations

While patients in this cohort were prospectively followed up for MACE, the study population was initially derived from a retrospective research database from a single tertiary hospital in the Republic of Korea. Notably, the patients included in this study had at least one confirmed or suspected cardiovascular condition who inherently have higher CVD risk than the general population. In this context, generalizability of the current study should be tested in a larger European population with diverse ethnic groups. Also, further research is needed to understand the joint association of Lp(a) level with well-established scores applicable to other populations such as SCORE2-OP and SCORE2-diabetes.^9,10^

In conclusion, our study suggests the potential importance of screening for Lp(a) levels among patients classified as low or moderate risk by ESC SCORE2. The findings of our study may provide additional insights on Lp(a)-targeted interventions to improve CVD prevention strategies.

## Data Availability

Due to privacy concerns and restrictions imposed by the committee of the Asan Medical Center (Seoul, Republic of Korea), the data used in this study are not made publicly available. Additional requests concerning this information should be addressed to the corresponding author.
